# Visualizing the Individual Arterial Anatomy of the Face Through Augmented Reality— A Useful and Accurate Tool During Dermal Filler Injections

**DOI:** 10.1093/asjof/ojac012

**Published:** 2022-02-18

**Authors:** Karl Waked, Marc Mespreuve, Joris De Ranter, Barbara Collard, Stephan Hahn, Benoit Hendrickx

## Abstract

**Background:**

The arterial anatomy of the face is extremely variable. Despite numerous cadaver dissections and anatomical descriptions, the exact location of the superficial facial arteries remains unpredictable. This ignorance is a determining factor in the pathophysiology of intravascular filler injections, potentially causing skin necrosis and blindness.

**Objectives:**

The main objective of this study is to evaluate the accuracy of an augmented reality (AR) application that visualizes the individual arterial anatomy of the face.

**Methods:**

A workflow was developed during which a magnetic resonance angiography (MRA) mapped the superficial arteries of the face. The images were further processed into an AR image that was visualized on the patient’s face using a specifically designed smartphone application. The accuracy of the AR image and the position of each individual artery were analyzed using duplex ultrasound (US).

**Results:**

A total of 216 facial arteries were visualized in 20 patients. The superficial temporal (100%), supratrochlear (92.5%), facial (75%), and angular (82.5%) arteries were visualized the most. The inferior labial (17.5%), dorsal nasal (22.5%), and supraorbital (42.5%) arteries were the most difficult to visualize through MRA. The average deviation between the artery visible on the AR image and the location assessed by US was 0.30 mm (standard deviation = +/− 0.66 mm). There were no complications reported.

**Conclusions:**

The combination of a risk-free MRA to map the individual arteries of the face and the processing into an AR image may be considered as a useful and accurate tool during dermal filler injections to potentially minimize the risk of intravascular filler injections.

The increase of augmented reality (AR) is one of the biggest breakthroughs of the 21st century. The use of AR in the modern society can be appreciated through multiple applications in the commercial world, the gaming industry, the architectural business, and then some.

It did not take too long before AR made its introduction into the medical world, with the first reports of AR in surgery dating back 25 years ago.^1^ In the same year, 1995, the first clinical case was described in which AR technology was used to accurately position dental implants.^2^

The core idea of AR—enhancing objects of the real world by computer-generated information—can truly be appreciated when looking at the patient’s own anatomy through AR. And for that, surgery is probably one of the most exciting playgrounds within that field. Allowing the surgeon to basically “see through the patient’s skin” is a real asset in surgical planning and execution. Although plastic surgeons are well trained in human anatomy, they are often confronted—and even more so fascinated—by the numerous anatomical variations that exist within the human body. For that reason, we perform preoperative imaging to visualize in detail the individual anatomy, the location of the perforators (in case of flap surgery), and the relationship between different tissues. The concept of anatomical variation—a term that generates more than 24,000 results on PubMed—is what makes surgery not only exciting/fascinating but also dangerous, and sometimes even frightening. The uncertainty of the exact anatomical location of a certain structure is one of the only certainties there is when stepping into the operating field. When dealing with anatomical variations, open surgery has a clear advantage over closed procedures as it allows the surgeon to analyze and meticulously dissect the tissues. However, when performing minimally or noninvasive operations, there is no clear view of the individual anatomy, and one must trust their theoretical (book) knowledge and the experience built up throughout the years.

With regard to dermal filler injections, knowledge of the arterial anatomy is of crucial importance, and the lack of these insights may lead to devastating complications such as skin necrosis and blindness.^3-7^ The pathway of intra-arterial hyaluronic acid (HA) injections has been described numerously.^8,9^ Unfortunately, possible solutions or surgical guidance tools to showcase each patient’s arterial network are still lacking.

This study may provide a possible workaround to deal with the uncertainty of the anatomical variation when performing filler injections. A previously described magnetic resonance angiography (MRA) imaging technique was used to map the superficial arterial network of the face (facial artery with its branches and the end branches of the ophthalmic artery).^10,11^ The obtained images provided the necessary information to develop an AR image that was visualized on the patient’s face. The projection of this arterial network was eventually checked by duplex ultrasound (US) in order to evaluate its accuracy with regard to the assumed location of each individual artery of the face. This workflow may potentially be the first breakthrough of the use of AR in the field of aesthetic and noninvasive surgery.

## METHODS

### Acquiring the MRA Images

The clinical study was approved by the ethical board of the hospital (AZ Zeno, Knokke, Belgium) with reference number 2020/14. The study was fully conducted at the hospital of AZ Zeno in Knokke (Belgium) and according to the principles of the declaration of Helsinki.

Only patients aged between 18 and 65 years were included in the study. Written consent was provided, by which the patients agreed to the use and analysis of their data. Scans were performed between March and May 2021. Exclusion criteria were non-MRI-compatible internal devices (such as a pacemaker), metal plates in the face or skull, dental braces (but not dental wires), tattoos on the face (but not permanent make-up), claustrophobia, vascular disease, and congenital or acquired facial anomaly. All included patients underwent a 3-dimensional (3D) Time of Flight Multiple Overlapping Thin Slab Acquisition (3D TOF MOTSA) MRA, of which the exact sequence has been published before.^11^ All images were acquired on a 3-Tesla (3-T) full-body magnetic resonance (MR) system, using a dedicated 21-channel head coil. As described previously, each patient was first positioned in the front of an infrared lamp to heat up the face before the MRA.^10-12^

### Processing the Digital Imaging and Communications in Medicine Images Into a 3D Volume

The native Digital Imaging and Communications in Medicine (DICOM) images of each patient were further processed to segment the arteries of the face. By using a segmentation and separation technique, a 3D volume of the superficial, subcutaneous (all arteries between the skin surface and the skull) arterial network was obtained. The dedicated algorithm was developed by Augmented Anatomy BV (Knokke, Belgium) using open-source libraries for medical imaging processing (Insight ToolKit  + Visualization ToolKit). A multiscale Jerman vessel enhancement function was applied on the MOTSA images to highlight arteries.^13^ Segmentation was performed by thresholding the vessel enhanced image. The 3D mesh was computed using the marching cubes method. The end results were exported into Standard Triangle Language (STL) volumes, which were then compared with the native DICOM images and rendered maximum intensity projection volume to evaluate which arteries were visualized by the MRA and further segmented into the 3D STL volume. A total of 18 arteries were taken into account, from both the left and right sides of the face: facial (Fa), superior (SL) and inferior labial (IL), angular (Ang), lateral nasal (LN), dorsal nasal (DN), superficial temporal (ST), supratrochlear (STr), and supraorbital (SO) arteries.

### Developing an AR Image of the Superficial Arteries of the Face

**Figure 1.**
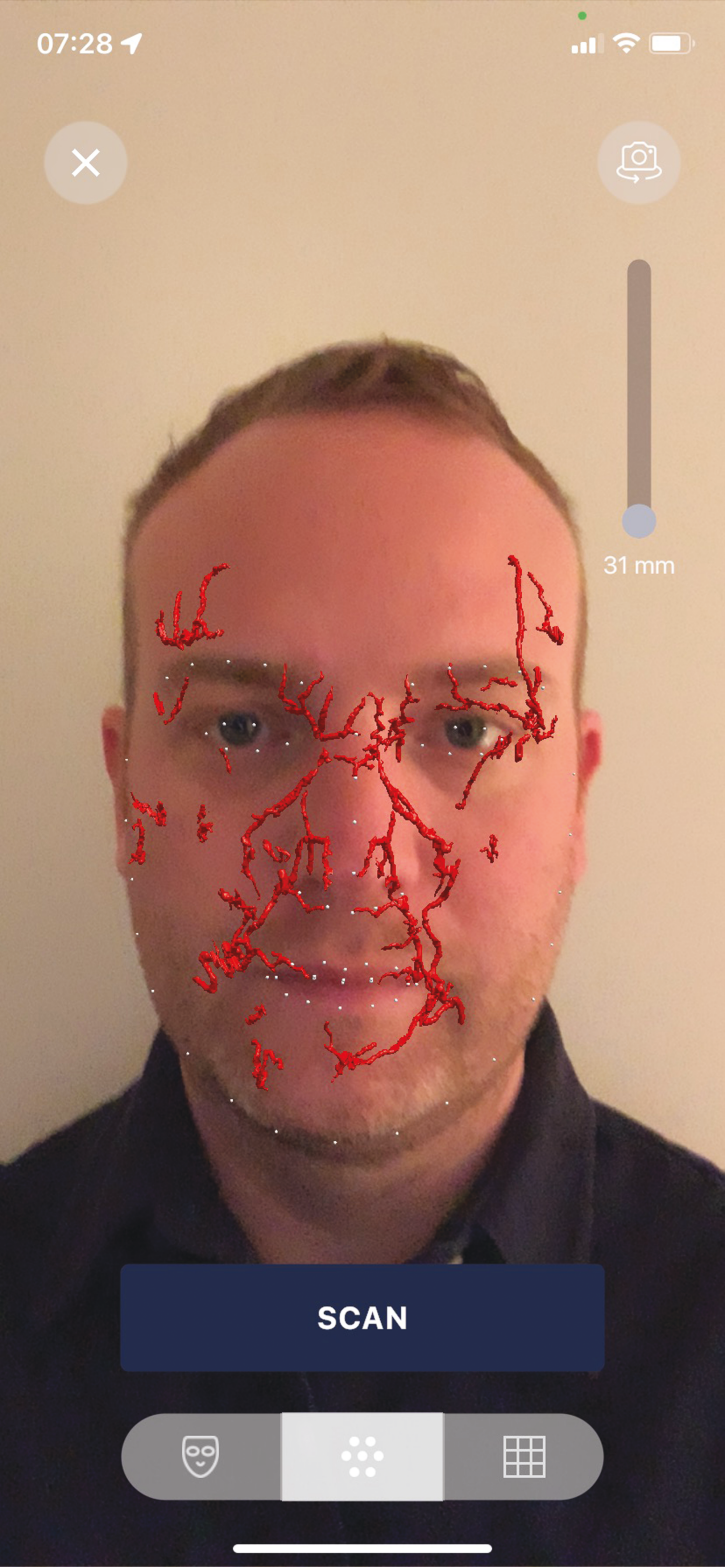
Visualization of the arterial anatomy of the face through augmented reality, based on the MRI of a 33-year-old male patient’s face.

To process the arterial network into an AR image, dedicated facial recognition software was used. The facial contour of each patient was scanned, and 68 unique reference points were determined. These were further matched according to the 3D volume of the arterial network. The reference points followed the contours of the jawline, chin, mouth, nose, eyes, and eyebrows. A smartphone application (Artery 3D by Augmented Anatomy, Knokke, Belgium) was used to visualize the AR image on each patient’s face. Using the smartphone’s back camera, the patient’s face was scanned and analyzed by the facial recognition software. The tracked reference points were then matched to the processed 3D arterial volume to accurately display the unique set of facial arteries on the patient’s face through AR. Figure 1 shows the facial tracking in a 33-year-old male patient.

### Analysis of the Accuracy of the AR Visualization of the Facial Arteries

**Figure 2.**
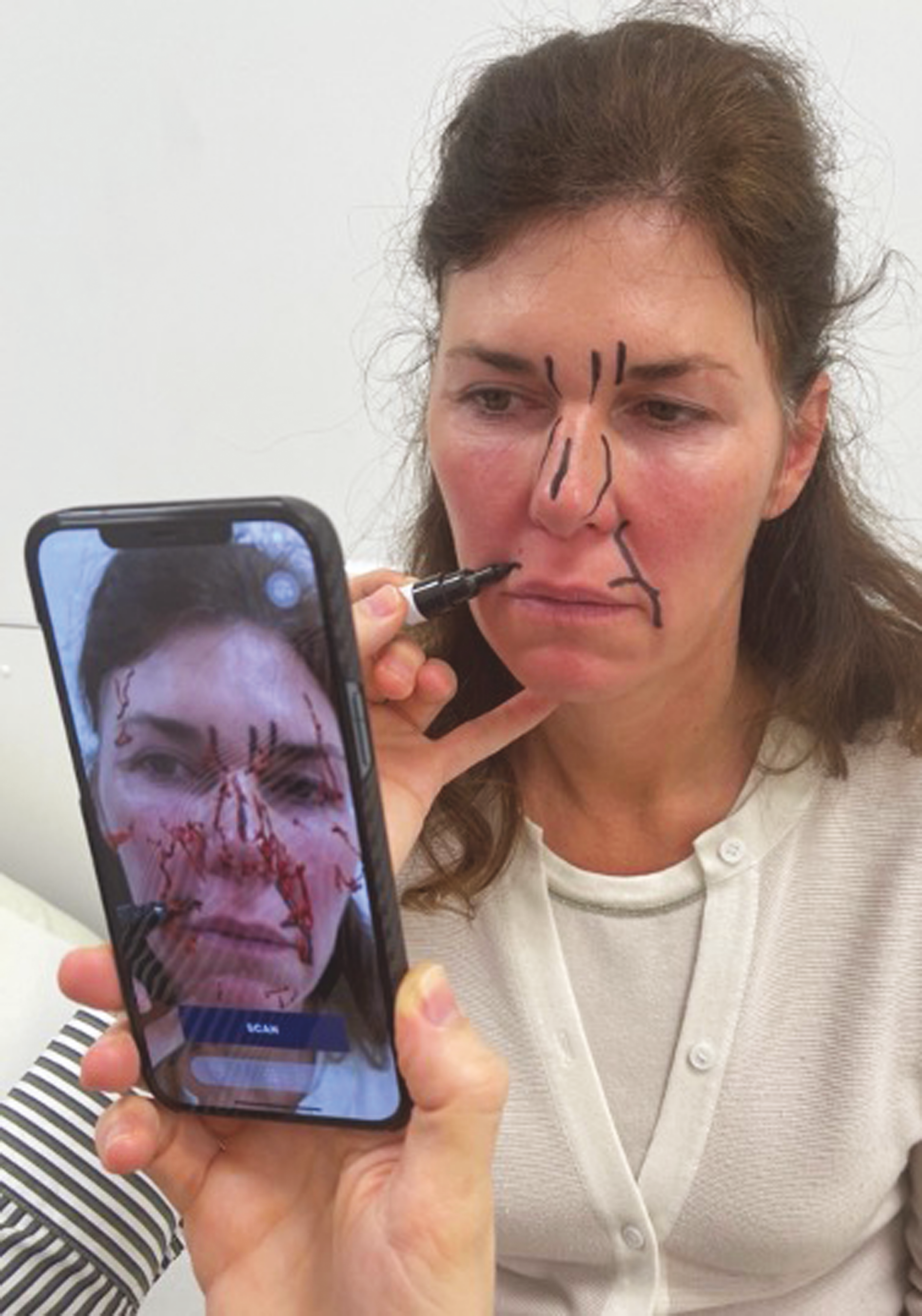
Drawing of the visualized superficial arteries of a 39-year-old female’s face using the Artery 3D application (Augmented Anatomy BV, Knokke, Belgium).

**Figure 3.**
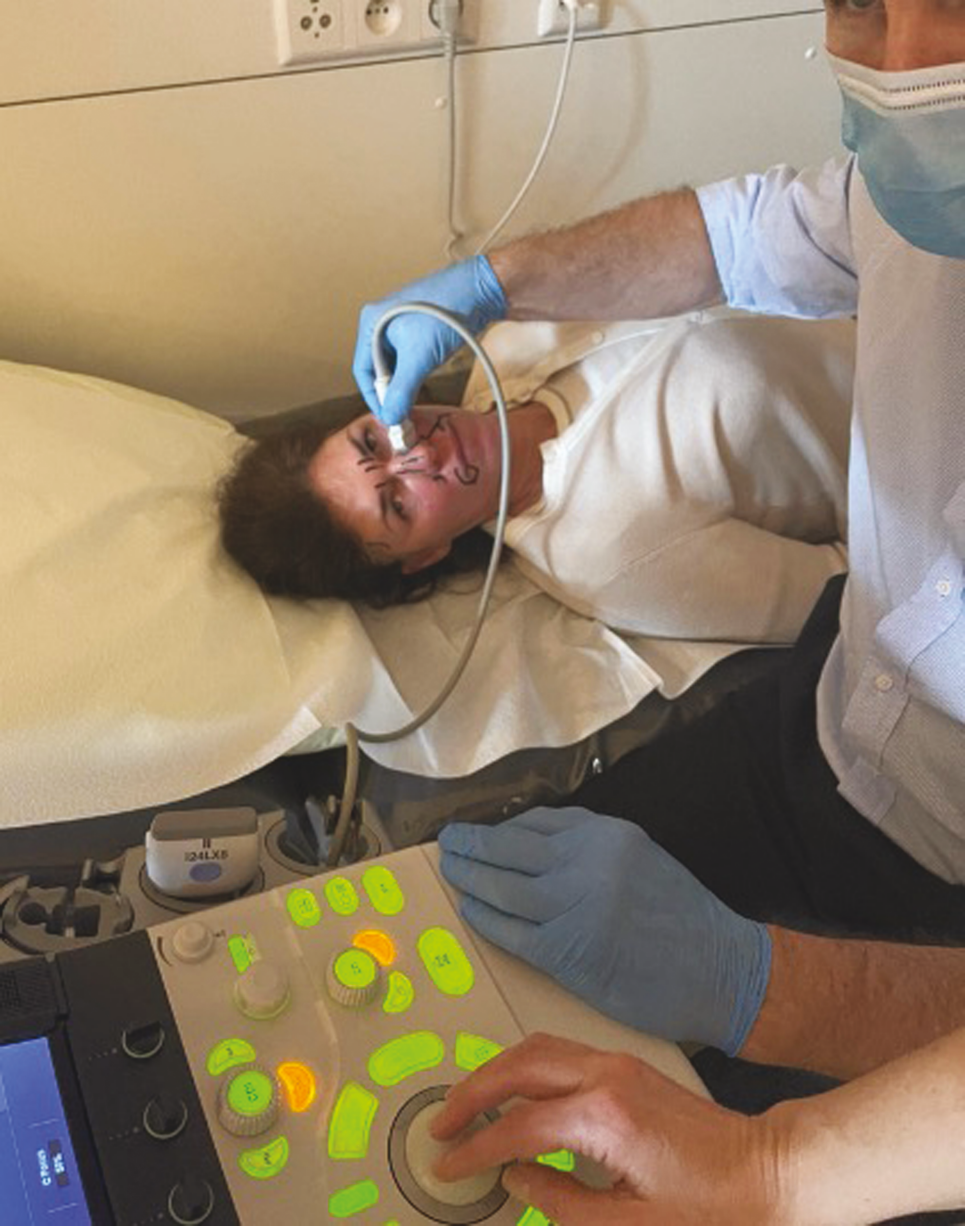
Determining the exact location of a superficial artery of the face using duplex ultrasound on a 39-year-old female.

**Figure 4.**
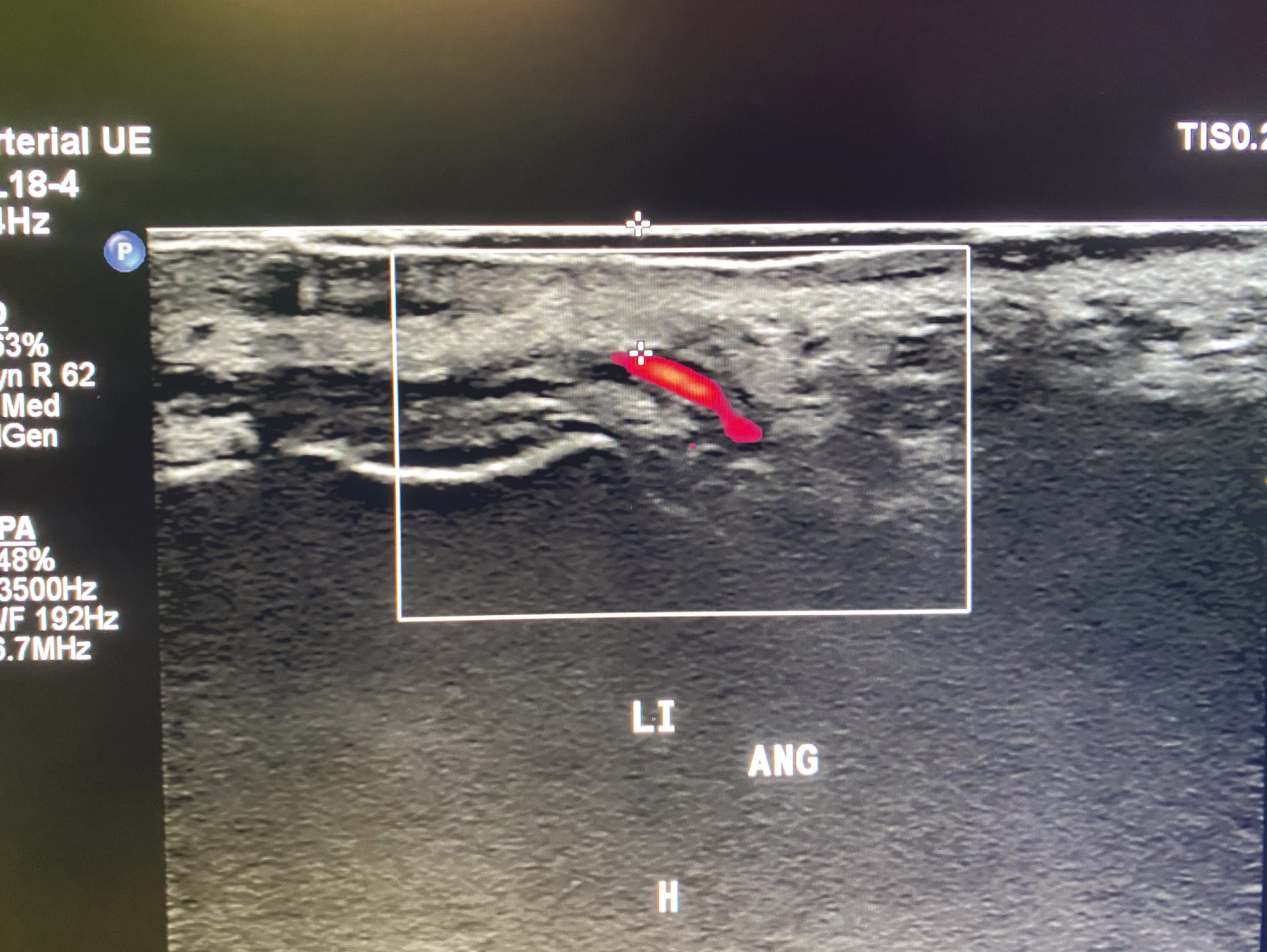
View of the left angular artery using duplex ultrasound. H, head; Li Ang, left angular artery.

**Figure 5.**
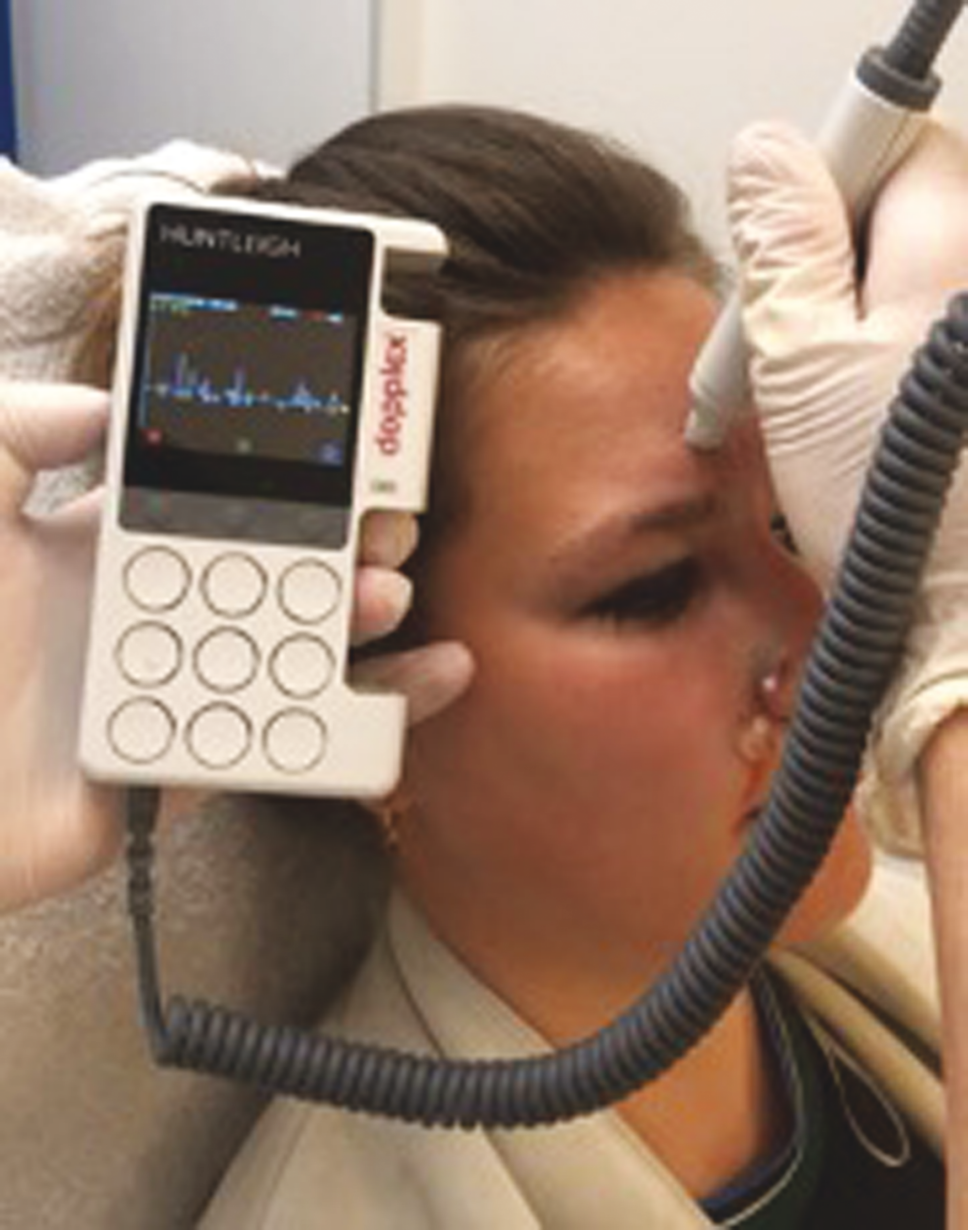
Determining the location of smaller superficial arteries of a 27-year-old female’s face by Doppler, which are not visualized by duplex ultrasound.

To analyze how accurate the arteries were positioned on the AR image, duplex US was used. The visualized arterial network was drawn on each patient’s face, using the AR projection as a guide (Figure 2). The exact position of each relevant facial artery was then determined by the duplex US, and the deviation between the AR projection and US location was recorded (Figures 3, 4). For each of the 18 relevant arteries (the total number of visualized arteries per patient), the average deviation was noted in millimeters. Before each US investigation, the patient was again placed in front of an infrared lamp for 15 minutes to induce vasodilation of the superficial arteries, thus rendering them more visible through the US. Arteries that were too small to visualize with the duplex US were located with US Doppler (Figure 5). Video shows a full overview of the developed technology and study setup. 

## RESULTS

Between March and May 2021, a total of 20 volunteers (mean age 46.7 and 10.1 years, range between 19 and 62 years) were recruited to map their individual arterial network of the face. Of the 20 patients, there were 4 males and 16 females. Dental braces or metal wires were present in 7 patients, resulting in metal artifacts for the superior and inferior labial arteries in 7 cases.

Each scan was processed into a 3D volume that was projected onto the patient’s face, using Artery 3D. Results were generated instantly, thanks to the automation of the segmentation and separation algorithm. Table 1 represents an overview of all the visualized superficial facial arteries on the 3D model, after segmentation and separation of the DICOM images. The most consistent visualized artery was the ST in 40/40 (100%) cases (left and right), followed by the Str in 37/40, (92.5%), the Ang in 33/40 (82.5%), the Fa in 30/40 (75%), and the LN in 25/40 (62.5%) cases. The IL and DN were the least visualized on MRA and on the rendered 3D volume in, respectively, 7/40 (17.5%) and 9/40 (22.5%) cases. In total, 216 (of the potential 360 arteries) superficial subcutaneous arteries of the face were visualized, of which 113 were on the left side and 103 on the right side of the face.

**Table 1.** Overview of the Visualized Superficial Facial Arteries on the 3-Dimensional Facial Model, After Segmentation and Separation of the Magnetic Resonance Angiography Images in 20 Patients

Artery	Number of arteries visualized (in 20 patients)			% of visualized arteries
	Left side	Right side	Total	Total
Facial	15	15	30	75.0%
Inferior labial	5	2	7	17.5%
Superior labial	10	8	18	45.0%
Angular	15	18	33	82.5%
Lateral nasal	14	11	25	62.5%
Dorsal nasal	4	5	9	22.5%
Supratrochlear	19	18	37	92.5%
Supraorbital	11	6	17	42.5%
Superficial temporal	20	20	40	100.0%
Total	113	103	226	

Table 2 presents an overview of the deviation (in millimeters) between the location of each artery, detected by the US, and the location drawn on the patient’s face, based on the projected AR image. The overall average deviation was 0.31 mm (standard deviation 0.66 mm) with an overall maximum deviation of 5 mm (in 1 case for the left superficial temporal artery) (see Table 3). The most accurate visualized artery was the IL, which was accurately visualized through the AR image in all 7 cases (Table 3). The AR visualization of the LN, DN, and STr was also quite accurate with an average deviation between the US location and AR location of 0.20 mm (+/− 0.58 mm), 0.22 mm (+/− 0.67 mm), and 0.22 mm (+/− 0.63 mm), respectively. The least accurate—albeit still quite precise within less than 1-mm average deviation—was the Fa (0.60 mm +/− 1.13 mm) and the ST (0.63 mm +/− 1.35 mm). We reported neither short- nor long-term complications, accounting both for the MRA investigation and for the US validation. 

**Table 2.** Overview of the Deviation (in millimeters) Between the Location of Each Artery, Illustrated by Duplex Ultrasound, and the Location Drawn on the Patient’s Face, Based on the Projected Augmented Reality Image of the Magnetic Resonance Angiography

Patient	Left side of the face									Right side of the face								
	Fa	IL	SL	Ang	LN	DN	STr	SO	ST	Fa	IL	SL	Ang	LN	DN	STr	SO	ST
1	0	0	0	0	0	—	0	—	5	0	—	—	0	0	0	0	—	3
2	2	—	—	—	0	—	0	—	0	—	—	—	0	0	—	—	—	0
3	0	0	—	0	—	—	0	—	0	0	0	0	0	0	—	—	—	0
4	3	—	—	—	—	—	2	2	0	3	—	—	—	—	—	0	0	0
5	0	—	—	0	0	—	0	—	0	0	—	—	0	0	—	0	—	0
6	3	—	0	3	0	—	0	0	0	2	—	—	0	—	—	0	—	0
7	3	—	0	2	0	—	0	0	0	2	—	3	2	0	—	0	0	0
8	0	0	0	1	0	—	2	0	0	0	—	—	0	1	—	2	0	0
9	0	—	0	0	0	—	0	—	4	0	—	0	0	0	—	0	—	0
10	0	—	0	0	0	—	0	0	0	0	—	0	0	0	—	0	0	0
11	—	—	—	—	0	—	0	—	0	0	—	—	0	0	—	0	—	0
12	0	—	0	2	2	0	0	0	0	0	0	0	2	—	0	0	—	0
13	—	—	0	0	2	—	0	0	0	—	—	—		—	—	0	—	0
14	—	—	—	—	—	2	0	0	2	0	—	0	0	—	—	0	0	2
15	—	0	0	—	—	0	0	0	4	—	—	0	2	—	0	0	—	2
16	0	—	—	0	—	0	2	2	0	0	—	—	0	—	—	0	0	0
17	0	—	—	0	0	—	0	—	0	0	—	—	0	0	—	0	—	0
18	0	0	0	0	0	—	0	—	3	0	—	2	0	0	—	0	—	0
19	0	—	—	0	0	—	0	0	0	—	—	—	0	—	0	0	—	0
20	—	—	—	0	—	—	—	—	0	—	—	—	0	—	0	0	—	0

“—” is used when the artery was not visible on the AR image. Ang, angular artery; DN, dorsal nasal artery; Fa, facial artery; IL, inferior labial artery; LN, lateral nasal artery; SL, superior labial artery; SO, supraorbital artery; ST, superficial temporal artery; STr, supratrochlear artery.

**Table 3.** Overview of the Mean, Maximum, and Minimum Average (in millimeters) Between the Location of Each Artery, Detected by Duplex Ultrasound, and the Location Drawn on the Patient’s Face, Based on the Projected Augmented Reality Image

	Left side of the face									Right side of the face									Bilateral									Total
	Fa	IL	SL	Ang	LN	DN	STr	SO	ST	Fa	IL	SL	Ang	LN	DN	STr	SO	ST	Fa	IL	SL	Ang	LN	DN	STr	SO	ST	
Number of visualized arteries	15	5	10	15	14	4	19	11	20	15	2	8	18	11	5	18	6	20	30	7	18	33	25	9	37	17	40	**216**
Mean average (mm)	0.73	0.00	0.00	0.53	0.29	0.50	0.32	0.36	0.90	0.47	0.00	0.63	0.33	0.09	0.00	0.11	0.00	0.35	0.60	0.00	0.28	0.42	0.20	0.22	0.22	0.24	0.63	**0.31**
Maximum average (mm)	3	0	0	3	2	2	2	2	5	3	0	3	2	1	0	2	0	3	3	0	3	3	2	2	2	2	5	**5**
Minimum average (mm)	0	0	0	0	0	0	0	0	0	0	0	0	0	0	0	0	0	0	0	0	0	0	0	0	0	0	0	**0**
Standard deviation (SD)	1.28	0.00	0.00	0.99	0.73	1.00	0.75	0.81	1.68	0.99	0.00	1.19	0.77	0.30	0.00	0.47	0.00	0.88	1.13	0.00	0.83	0.87	0.58	0.67	0.63	0.66	1.35	**0.66**

“Number of visualised arteries” = the number of patients in which a specific artery was visualised on the MRI and augmented reality (AR) image; “mean average” = the mean average deviation between the AR-location of an artery and the location determined by ultrasound (US); “maximum average” = the maximum deviation between the AR-location of an artery and the location determined by ultrasound (US) - “minimum average” = the minimum deviation between the AR-location of an artery and the location determined by ultrasound (US).

Ang, angular artery; DN, dorsal nasal artery; Fa, facial artery; IL, inferior labial artery; LN, lateral nasal artery; SL, superior labial artery; SO, supraorbital artery; ST, superficial temporal artery; STr, supratrochlear artery.

## DISCUSSION

Neurosurgery is probably the number one player in the field of AR-guided surgery and has truly embraced the use of this exciting new technology in its day-to-day surgical planning.^14^ Within the field of plastic and reconstructive surgery, the use of AR has been described for a number of procedures but has yet to find a solid base to be implemented in the plastic surgeon’s daily practice. Some authors have described its use in the visualization of the arterial anatomy during preoperative flap planning, while others found a benefit in AR-guided flap volume visualization.^15-18^ One of the most interesting applications was described by Pratt et al in 2018, in which the Microsoft HoloLens (Redmond, WA) was used to visualize perforating vessels of the lower limb through AR.^19^ The AR visualization was generated from the patient’s own computed tomography angiography (CTA) images and allowed the surgeon to accurately locate the perforating vessels of the vascular trunk of the lower limb. A more “sophisticated” implementation of an intraoperative AR system has been published 2 years later by Katayama et al.^20^ One of the most popular free flaps in reconstructive surgery is the deep inferior epigastric artery perforator (DIEAP) flap. Aiding the surgeon by means of an AR-based system to accurately locate the perforator vessels and their intramuscular course may result in better operation planning, faster flap dissection times, and less donor site morbidity, eventually leading to better postoperative outcomes.^15,17,21^ Even though not fully embraced yet, these early reports are a perfect example of why our discipline would profit from a kind of technology that lets you “see through the patient’s skin” using the newest and state-of-the-art 3D and AR visualization systems.

Demanding any kind of invasive or potential harmful investigation like a CTA or classic angiography in aesthetic cases would be unethical and completely unjustifiable. However, taking into account the ever-increasing reports on anatomical variation, as well as those portraying vascular-related complications of dermal filler products, there is a need to help the injector to locate potential facial danger zones.^3-6,8,22–25^

In 2016, the Global Aesthetic Consensus Group published a list of recommendations to potentially prevent and manage complications related to HA injections.^24^ Related to vascular occlusions, the recommendations entail a thorough knowledge of the vascular anatomy, adequate training, and appropriate injection technique. Knowledge of the vascular anatomy is a necessity. However, the numerous described variations of almost every relevant superficial artery of the face will undoubtedly cause some level of uncertainty for even the highest trained injectors.^3,23,25^ Regarding appropriate techniques, it is recommended to aspirate before injecting any amount of HA and to use blunt microcannulas to minimize the risk of vessel wall perforation.^24^ However, even those 2 widely proclaimed “safety measures” do not seem waterproof and cannot ensure enough safety during filler injections. Van Loghem et al. published a detailed report in 2018, showing that aspiration tests are heavily influenced by several variables, such as needle diameter, needle length, and rheological properties of soft tissue fillers (STF).^26^ The longer and narrower the needle of the filler syringe, the more false-negative (ie, no aspiration possible of the STF) results were observed, due to a higher flow resistance during aspiration. The same accounts for the rheological properties of the used STF, where high cohesive products will often give negative aspiration test results. Only one-third (112/340) of the aspiration tests were positive within 1 second of aspiration, whereas 37.6% of the tests still were false-negative after 10 seconds of aspiration. Finally, 30% of the tests were positive when aspirating between 1 and 10 seconds. In conclusion, aspirating for at least 10 seconds seems mandatory, but even then, only 63% (213/340) of the tests were true-positive. Reports focusing on cannula size also claim that cannulas smaller than 25 gauge behave like needles within the human body and can easily puncture a vascular wall when enough force is applied.^27,28^

Multiple algorithms have been published to treat the patient in case of an STF-induced vascular occlusion.^24^ The cornerstone remains abundant use of hyaluronidase to dissolve the HA when the injector fears eminent skin necrosis or retinal infarction. However, although highly recommended and lifesaving in several case reports, its success remains doubtful in the case of retinal artery occlusion.^29^

Without a doubt, the best way to treat complications is by preventing them. In the case of STF-induced vascular occlusion, an accurate visualization of the individual arterial anatomy may be useful, as it eliminates the uncertainties when trusting solely the standard anatomical knowledge. In our opinion, the previously described MRA sequence is the only published imaging method to date that provides an all-in-one overview of the relevant arterial anatomy for any injector.^10,12^

The sensitivity of this MRA sequence was already evaluated in a previous study and is beyond the scope of this article.^10^ However, it is interesting to notice that the developed MRA sequence results in a much more complete visualization of the facial arterial network, when performed on a 3-T MRI. Compared with our first study (which was performed on a 1.5-T MRI), we have an overall far better visualization of the facial arteries, and more in particular of the Fa (75.0%), Ang (82.5%), ST (100.0%), and Str (92.5%) arteries.^10^ As these arteries are located in the riskiest zones with regard to vascular filler complications (ie, nasolabial fold for the Fa and Ang, temporal fossa for the ST, and glabellar region for the Str), the importance of their visualization—and more importantly, their anatomical variation—cannot be underestimated. If one would exclude the patients who wore dental braces (7 of the 20) for the statistical analysis of the labial arteries, the sensitivity of the MRA sequence would increase from 17.5% and 45.0% to 26.9% and 69.2% for the IL and SL, respectively. Moreover, with a mean average deviation of less than 1 mm between the AR location and US location—as proven by this study—we are quite confident about the clinical importance of the developed AR application.

The major downside of MRA remains the metal artifacts, which are known to disturb the image. As the population that wears or wore braces, often followed by dental wires, is quite abundant, we do realize that the visualization of the labial arteries will be very variable and dependent of the examined patient population. Despite these shortcomings, an MRI-based workflow remains the only imaging technique that provides a risk-free and noninvasive 3D overview of the facial vasculature. US-based systems have been shown to provide some level of guidance during STF injections.^30,31^ However, the added technical complexity of visualizing small vascular structures as a non-radiologist, the dexterity that is needed to hold the probe in one hand and inject an STF with the other, and the issues of sterility when guiding the needle through the conductive gel do seem severe limitations in the practical implementation of this guidance system.

We do realize that there is a margin of error in the presented workflow. Both the process of drawing the arteries on the face and determining the US-guided location of each artery may result in a deviation between the assumed and real arterial location. Additionally, the used marker to draw the arteries has a thickness of 1 mm, which may influence the measurements as well. Deviations for each individual artery ranged between 0 and 3 mm, with only the ST artery deviating twice 4 mm (in patients 9 and 15) and once 5 mm (patient 1).

However, by including well over 200 individual arteries, we assume that the calculated average deviations provide a quite trustful picture of the levels of accuracy of the developed application. Moreover, we especially chose to use the duplex US to eliminate possible arterial signals of a small, more superficial arterial branch that may be confounded with the relevant and targeted artery.

The current version of the AR visualization method does not take depth into account. It is true that the arteries run at different levels within the subcutaneous tissue and that depth information is crucial when injecting filler products. Showing the depth of each artery (either by pointing out the distance between the artery and skin or by developing a true 3D visualization of the application for AR glasses) may certainly bring added value to this workflow and will hopefully be part of future software updates. In addition, the use of AR glasses will further improve the accurate tracking of the facial contours, hence further increasing the accuracy of the AR visualization of the facial arteries.

## CONCLUSIONS

The world of STF injections is a massively booming business. The number of injectors increases day by day and so does the number of complications. Although measurements such as knowledge of the anatomy, correct injection techniques, and point-by-point treatment algorithms may provide some level of security, it is only by knowing the exact location of the facial arteries that filler-induced vascular occlusions may truly be avoided. This study proves that not only the developed MRA sequence is able to map out the most relevant superficial arteries of the face and help the injector in analyzing the facial danger zones, but also the developed AR application can additionally provide an accurate (up to 0.1 mm) and real-time visualization of the individual anatomical variations of each patient. In these exciting times of technological innovation, evolutions in the field of augmented and virtual reality may hopefully become a part of the injector’s armamentarium in the near future. After all, it is the patient’s safety that remains the cornerstone of any medical or surgical treatment, and we should embrace any innovation or development that strives toward that uniform goal.
